# Achado Incomum de Rara e Exuberante Xantomatose em Caso de Hiperlipidemia

**DOI:** 10.36660/abc.20200999

**Published:** 2021-08-09

**Authors:** Enrico Manfredini, Renato Jorge Alves

**Affiliations:** 1 Faculdade de Ciências Médicas da Santa Casa de São Paulo São PauloSP Brasil Faculdade de Ciências Médicas da Santa Casa de São Paulo (FCMSCSP), São Paulo, SP - Brasil; 2 Irmandade da Santa Casa de Misericórdia de São Paulo Departamento de Medicina São PauloSP Brasil Irmandade da Santa Casa de Misericórdia de São Paulo - Departamento de Medicina, São Paulo, SP – Brasil.

**Keywords:** Hiperlipidemias, Dislipidemias, Xantomatose, Hipolipemiantes

## Introdução

Hiperlipidemias podem aumentar a morbimortalidade. Foram classificadas por Fredrickson em fenótipos: I, IIa, IIb, III, IV e V.^1^^–^^5^ Nas mistas, há hipercolesterolemia e hipertrigliceridemia, fenótipos IIb e III, com colesterolemia e trigliceridemia de 250 a 300 mg/dL no fenótipo IIb, e 500 a 600 mg/dL ou mais, no III, respectivamente. É incomum o aparecimento de pancreatite em ambos, bem como xantomatose no IIb. Xantomas e complicações cardiovasculares são mais frequentes no fenótipo III.^2^^,^^6^

Apresentamos um caso de hiperlipidemia com relevantes alterações lipídicas, pancreatite e exuberante xantomatose.

## Relato do Caso

Masculino, 48 anos de idade, natural de Manaus, comerciante, com histórico de pancreatite hemorrágica (2004), hipertensão arterial e diabetes tipo 2 desde 2006, retinopatia hipertensiva grau 3 e retinopatia diabética proliferativa grave. Negava tanto histórico familiar de doenças cardiovasculares ou dislipidemias quanto consanguinidade na família. Usava enalapril 10 mg/dia, dapagliflozina 5 mg/dia, metformina 1.000 mg/dia, gliclazida 120 mg/dia e insulina NPH 16UI/dia. Negava uso prévio de estatina, apenas fibrato irregularmente.

Assintomático e anictérico. Peso: 89 kg, altura: 172 cm, IMC: 30,1 kg/m^2^, pressão arterial: 120/90 mmHg, frequência cardíaca: 80 bpm. Pulmões limpos, bulhas rítmicas normofonéticas, sopro protossistólico em área aórtica 2/6+, sem sopros carotídeos. Pulsos distais regulares e sem alterações. Abdome globoso, com cicatriz xifoumbilical. Membros inferiores sem edema.

**Figura 1:**
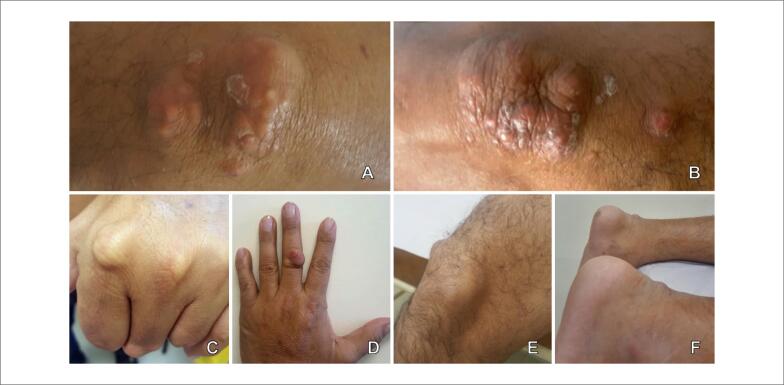
Xantomatose prévia. A) Cotovelo direito. B) Cotovelo esquerdo. C) Segunda articulação metacarpofalângica esquerda. D) Terceira articulação interfalângica proximal direita. E) Joelho direito. ) Ambos os tendões de Aquiles. Fonte: imagens obtidas pelos autores durante consulta de rotina.

Destacava-se a presença de múltiplas e extensas lesões nodulares, indolores, em cotovelos, articulações metacarpofalângicas e interfalângicas bilateralmente, joelhos e tornozelos, compatíveis com xantomas tuberosos e tendinosos (Figura 1). Não apresentava xantoma estriado palmar.

Foi realizada eletroforese de lipoproteínas: fração alfa 6,2%, beta e pré-beta 93,8%, compatível com fenótipo IIb.

**Figura 2:**
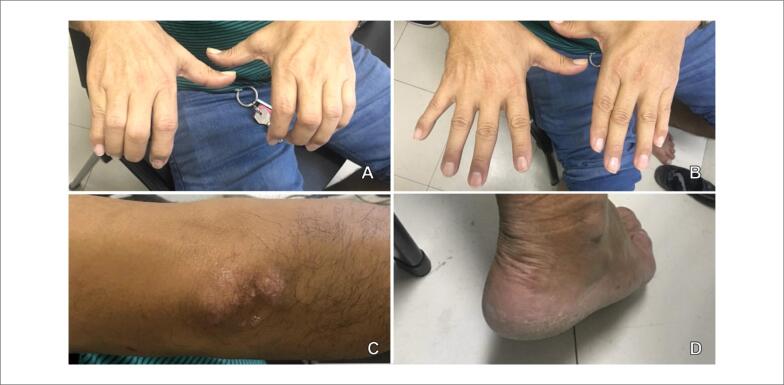
Regressão dos xantomas. A e B) Articulações interfalângicas e metacarpofalângicas. C) Cotovelo direito. D. Região do tendão de Aquiles direito. Fonte: imagens obtidas pelos autores durante consulta de rotina.

Devido ao quadro da hiperlipidemia, optou-se pela introdução de atorvastatina 40, após 6 meses, 80 mg/dia e ciprofibrato 100 mg/dia, associados a modificações do estilo de vida e dietoterapia. Após essa terapêutica, houve regressão significativa das lesões xantomatosas (Figura 2) e da hiperlipidemia (Tabela 1).

**Tabela 1:** Exames laboratoriais

Exames laboratoriais	Anterior ao tratamento	Após tratamento
Triglicérides	2.407 mg/dL	291 mg/dL
Colesterol total	513 mg/dL	144 mg/dL
HDL-c	40 mg/dL	36 mg/dL
LDL-c	NC	50 mg/dL
Glicemia	234 mg/dL	137 mg/dL
Hemoglobina glicada	10%	7,1%
TGO	12 U/L	13 U/L
TGP	21 U/L	7 U/L
TSH	4,27 mU/L	3,62 mU/L
CPK	VI	74 U/L
Creatinina	0,7 mg/dL	VI
Ácido úrico	VI	6,4 mg/dL

HDL: lipoproteína de alta densidade; LDL: lipoproteína de baixa densidade; NC: não calculado pela Fórmula de Friedewald; VI: valor indisponível; TGO: aspartato aminotransferase; TGP: alanina aminotransferase; TSH: hormônio estimulante da tireoide; CPK: creatinofosfoquinase. Fonte: revisão de prontuário pelos autores.

## Discussão

Trata-se de achado incomum de xantomatose difusa em paciente com fenótipo IIb, que costuma migrar para IIa e IV na prática clínica.

Essa xantomatose é raramente vista no fenótipo IIb, principalmente a forma tuberosa em tendão de Aquiles, mais encontrada em hipercolesterolemia familiar (HF).^7^ O ecocardiograma evidenciou calcificação da válvula aórtica, achado também em casos graves de HFou de elevação plasmática de lipoproteína(a) (Lp[a]).^8^^,^^9^ No entanto, uma resposta muito satisfatória à terapia com estatina, como ocorreu nesse caso, não seria comum na HF, principalmente na forma homozigótica.^1^^,^^3^

A hipertrigliceridemia acentuada indicaria fenótipo IV ou V; contudo, a eletroforese de lipoproteínas mostrou elevações das frações beta e pré-beta.^6^ Entretanto, hipertrigliceridemia >1.500 mg/dL com xantomas tuberosos e tendinosos seria compatível com dislipidemia mista.^2^^,^^6^

No fenótipo III, além de xantomatose tuberosa e eruptiva, haveria xantomatose palmar e doença aterosclerótica precoce.^9^ Ainda, as concentrações plasmáticas de colesterol e triglicérides seriam muito elevadas, mas quase similares. Entretanto, pelo fato de os distúrbios metabólicos terem contribuído para o agravamento do quadro clínico e os xantomas se assemelharem à xantomatose tuberoeruptiva, disbetalipoproteinemia (tipo III) associada a defeitos genéticos, como HF ou elevação de Lp(a), seria a hipótese apropriada a ser considerada.

Outras hipóteses excluídas seriam: xantomatose cerebrotendinosa – não há alterações neurológicas,^10^^,^^11^ e sitosterolemia, devido à resposta satisfatória com estatina,^12^^,^^13^ embora pudesse ser excluída por genotipagem.

É relevante relatar a ocorrência de pancreatite aguda em 2004, com consequente diabetes, mais frequente no fenótipo I que no V.^2^^,^^6^^,^^14^ Nesse caso, o diagnóstico de diabetes se deu após o relato de pancreatite, sugerindo hipertrigliceridemia relevante, por causa genética ou ambiental, pois o paciente não era totalmente aderente ao uso de fibrato.

Nem sempre o fenótipo das dislipidemias mostra-se claro, mesmo com exames complementares, dificultando o diagnóstico precoce e a conduta apropriada.^15^

A associação de estatina (alta potência) e ciprofibrato alcançou o objetivo esperado, haja vista os resultados laboratoriais e a cicatrização dos xantomas. Caso não fossem alcançadas as metas de lipoproteína de baixa densidade (LDL-c), a associação de estatina com inibidores de pró-proteína convertase subtilisina/kexina tipo 9 (PCSK9) ou ezetimiba seria uma opção, assim como ômega-3, em conjunto com fibrato, para redução da hipertrigliceridemia.^2^

Como limitações, ressaltamos que, por indisponibilidade na instituição, não foram realizados: angiotomografia de coronárias, para melhor estratificar o risco cardiovascular,^2^^,^^16^ apesar de o paciente ser de alto risco,^9^ e testes genéticos, para avaliar possíveis mutações em lipase lipoproteica e apolipoproteína E. Apesar disso, a avaliação clínica e laboratorial, aliada à experiência do serviço, foi fundamental para o resultado satisfatório, evitando-se manifestação de desfecho aterosclerotíco ou de nova pancreatite. Embora cada vez mais presente, a genotipagem ainda não está amplamente disponível em muitos países e serviços.^17^^,^^18^

## Conclusão

Mesmo com as dificuldades inerentes à investigação laboratorial, a *expertise* em detectar e tratar adequadamente caso raro e grave de dislipidemia foi fundamental para a melhora laboratorial e a prevenção de resultados clínicos potencialmente fatais.
