# Supplementary material for: Achado Incomum de Rara e Exuberante Xantomatose em Caso de Hiperlipidemia
Source: Arq Bras Cardiol. 2021 Aug 9;117(2):407–10. [Article in Portuguese] doi: 10.36660/abc.20200999 (PMC8395804; doi:10.36660/abc.20200999)
Supplement: Translation

# Translation: Unusual Finding of Rare Exuberant Xanthomatosis in Hyperlipidemia

**Authors:** Enrico Manfredini, Renato Jorge Alves

**Affiliations:** 1 São PauloSP Brazil Faculdade de Ciências Médicas da Santa Casa de São Paulo (FCMSCSP), São Paulo, SP - Brazil.; 2 São PauloSP Brazil Irmandade da Santa Casa de Misericórdia de São Paulo - Departamento de Medicina, São Paulo, SP – Brazil.

**Keywords:** Hyperlipidemias, Dyslipidemias, Xanthomatosis, Hypolipidemic Agents

## Introduction

Hyperlipidemia can increase morbidity and mortality. This condition has been classified by Fredrickson into the following phenotypes: I, IIa, IIb, III, IV and V.^1^^–^^5^ In the mixed ones, the following conditions are present: hypercholesterolemia and hypertriglyceridemia, phenotypes IIb and III, with cholesterolemia and triglyceridemia of 250 to 300 mg/dL in phenotype IIb, and 500 to 600 mg/dL or more, in III, respectively. Pancreatitis is uncommon in both, as well as xanthomatosis in IIb. Xanthomas and cardiovascular complications are more frequent in phenotype III.^2^^,^^6^

We present a case of hyperlipidemia with relevant lipid abnormalities, pancreatitis and exuberant xanthomatosis.

## Case Report

Male, 48 years old, born in Manaus, retailer, with a history of hemorrhagic pancreatitis (2004), arterial hypertension and type 2 diabetes since 2006, grade 3 hypertensive retinopathy and severe proliferative diabetic retinopathy. Denied family history of cardiovascular disease or dyslipidemia and denied consanguinity in the family. Used enalapril 10 mg/day, dapagliflozin 5 mg/day, metformin 1000 mg/day, gliclazide 120 mg/day and NPH insulin 16 UI/day. Denied previous use of statin, only fibrate irregularly.

Asymptomatic and anicteric. Weight: 89 Kg, height: 172 cm, BMI: 30.1 kg/m^2^, blood pressure: 120/90 mmHg, heart rate: 80 bpm. Clean lungs, normophonetic rhythmic heart sounds, protosystolic murmur in aortic area 2/6+, no carotid murmurs. Regular and unaltered distal pulses. Distended abdomen with umbilical scar. Lower limbs with no edema.

Presence of multiple extensive painless nodular lesions in the elbows, bilateral metacarpophalangeal and interphalangeal joints, knees and ankles, compatible with tuberous and tendinous xanthomas (Figure 1). No striated palmar xanthoma.

**Figure 1:** Previous xanthomatosis: A) Right elbow; B) Left elbow; C) Second joint left metacarpophalangeal; D) Third right proximal interphalangeal joint; E) Right knee; F) Both Achilles tendons. Source: images taken by the authors at a routine appointment.

Lipoprotein electrophoresis was performed: alpha fraction 6.2%, beta and pre-beta 93.8%, compatible with phenotype IIb.

Due to hyperlipidemia, it was decided to introduce atorvastatin 40, after 6 months, 80 mg/day, and ciprofibrate 100 mg/day, associated with lifestyle modifications and diet therapy. After this therapy, there was a significant regression of xanthomatous lesions (Figure 2) and hyperlipidemia (Table 1).

**Figure 2:** Regression of xanthomas: A and B) Interphalangeal and metacarpophalangeal joints; C) Right elbow; D) Right Achilles tendon region. Source: images taken by the authors at a routine appointment.

**Table 1:** Laboratory tests

Laboratory tests	Before treatment	After treatment
Triglycerides	2407 mg/dL	291 mg/dL
Total cholesterol	513 mg/dL	144 mg/dL
HDL-c	40 mg/dL	36 mg/dL
LDL-c	NC	50 mg/dL
Glucose	234 mg/dL	137 mg/dL
Glycated hemoglobin	10%	7,1%
GOT	12 U/L	13 U/L
ALT	21 U/L	7 U/L
TSH	4.27 mU/L	3.62 mU/L
CPK	VI	74 U/L
Creatinine	0.7 mg/dL	VI
Uric acid	VI	6.4 mg/dL

HDL: High-density lipoprotein; LDL: low-density lipoprotein; NC: not calculated by Friedewald’s Formula; VI: unavailable; GOT: glutamic oxaloacetic transaminase; ALT: alanine transaminase; TSH: thyroid stimulating hormone; CPK: creatine phosphokinase. Source: review of medical records by the authors.

## Discussion

This is an uncommon finding of diffuse xanthomatosis in a patient with the IIb phenotype, which usually migrates to IIa and IV in clinical practice.

This xanthomatosis is rarely seen in the IIb phenotype, especially the tuberous form in the Achilles tendon, more commonly found in familial hypercholesterolemia (FH).^7^ Echocardiogram showed calcification of the aortic valve, also found in severe cases of FH or lipoprotein plasma elevation(a) – Lp(a).^8^^,^^9^ However, a very satisfactory response to statin therapy, as occurred in this case, would not be common in FH, especially in the homozygous form.^1^^,^^3^

Marked hypertriglyceridemia would indicate phenotype IV or V; however, lipoprotein electrophoresis showed elevations in beta and pre-beta fractions.^6^ However, hypertriglyceridemia >1500 mg/dL with tuberous and tendinous xanthomas would be compatible with mixed dyslipidemia.^2^^,^^6^

In phenotype III, in addition to tuberous and eruptive xanthomatosis, there would be palmar xanthomatosis and early atherosclerotic disease.^9^ Furthermore, plasma concentrations of cholesterol and triglycerides would be very high, but almost similar. However, because the metabolic disorders have contributed to worsening of the condition and the xanthomas resemble tuberoeruptive xanthomatosis, dysbetalipoproteinemia (type III) associated with genetic defects such as FH or Lp(a) elevation, it would be the appropriate hypothesis to be considered.

Other ruled out hypotheses would be: cerebrotendinous xanthomatosis, no neurological alterations,^10^^,^^11^ and sitosterolemia, due to a satisfactory response with statin,^12^^,^^13^ although it could be ruled out by genotyping.

It is relevant to report the occurrence of acute pancreatitis in 2004, with consequent diabetes, more frequent in phenotype I than in V.^2^^,^^6^^,^^14^In this case, diagnosis of diabetes occurred after report of pancreatitis, suggesting relevant hypertriglyceridemia, due to genetic or environmental causes, as the patient was not fully following the fibrate therapy.

The dyslipidemia phenotype is not always clear, even with complementary tests, making difficult to deliver early diagnosis and conduct appropriate management.^15^

Association of statin (high potency) and ciprofibrate achieved the expected objective, as seen in the laboratory results and the healing of xanthomas. If low-density lipoprotein (LDL-c) targets were not met, the association of statin with proprotein convertase subtilisin/kexin type 9 (PCSK9) inhibitors or ezetimibe would be an option, as would omega-3 with fibrate to reduce hypertriglyceridemia.^2^

As limitations, due to unavailability in the institution, we did not perform: coronary angiotomography to better stratify cardiovascular risk,^2^^,^^16^ although the patient is at high risk,^9^ and genetic tests to assess possible mutations in lipoprotein lipase and apolipoprotein E. Despite this, clinical and laboratory evaluation combined with the experience of the service were essential for a satisfactory result, avoiding an atherosclerotic outcome or a new pancreatitis. Although increasingly present, genotyping is not widely available in many countries and services.^17^^,^^18^

## Conclusion

Despite the difficulties found in laboratory investigation, expertise in detecting and adequately treating a rare and severe case of dyslipidemia was essential for laboratory improvement and to prevent potentially fatal clinical outcomes.
